# 1248. Relationship Between ALT and New HBV Biomarkers in HBV/HIV Coinfected Persons Started on Antiviral Therapy

**DOI:** 10.1093/ofid/ofac492.1079

**Published:** 2022-12-15

**Authors:** Susan D Rouster, Jason T Blackard, Paul S Horn, Michael E Stec, Kenneth E Sherman, Mark C Anderson, Marion G Peters, Gavin Cloherty

**Affiliations:** University of Cincinnati, Cincinnati, Ohio; University of Cincinnati College of Medicine, Cincinnati, Ohio; Cincinnati Children's Hospital Medical Center, Cincinnati, Ohio; Abbott Laboratories, Abbott Park, Illinois; University of Cincinnati, Cincinnati, Ohio; Abbott Laboratories, Abbott Park, Illinois; Northwestern University, San Francisco, California; Abbott, Abbott Park, Illinois

## Abstract

**Background:**

While HBV DNA has served as a primary biomarker of HBV infection, newer biomarkers including pre-genomic HBV RNA (pgRNA) and quantitative HBV surface antigen (qHBsAg) may prognosticate treatment responses better. We analyzed the changes in these biomarkers over time following initiation of antiviral therapy in persons living with HIV (PLWH) to determine the relationship to ALT, a key marker of liver injury.

**Figure:**
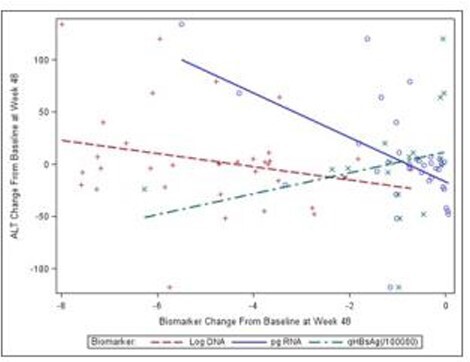
Biomarker changes

**Methods:**

NIH AIDS Clinical Trials Group Study 5127 enrolled HBV/HIV PLWH into a Phase 2 treatment trial that randomized them to receive 48 weeks of either tenofovir (TDF) or adefovir (ADV). ALT and HBV DNA were evaluated as measures of treatment response. Stored samples were tested for HBV pgRNA and qHBsAg at baseline, 4, 12, 24, 36, and 48 weeks. HBV pgRNA was isolated using RNA-selective extraction chemistry followed by a multiplex RT-qPCR that targets the HBV X and core targets on the m2000 system (Abbott Molecular, Des Plaines, Illinois). A sensitive second-generation assay with a lower limit of quantification (LLOQ) of 10 copies/mL was utilized. Quantitative HBsAg was determined by Abbott ARCHITECT (List# 6C36) with a LLOQ of 0.05 IU/mL. Data were analyzed using SAS® version 9.4.

**Results:**

Overall, 47 study participants had sufficient samples for biomarker testing. Participants were predominantly male (93.6%) and identified as white, non-Hispanic (55.3%) or black (34%). Groups were evenly randomized to receive either TDF (n=23) or ADV (n=24). Baseline ALT mean was 64.9 U/L, and HBV DNA was 9.1 (+1.6) copies/mL. Pre-treatment pgRNA serum concentration was 7.0 (+1.3) log U/mL, and qHBsAg was 195,574 (+272,190) IU/mL. Over the course of treatment, pgRNA was significantly correlated with the change in serum ALT levels (r=0.48; p=0.0035) but not HBV DNA (r=0.21; p=0.226). ALT was negatively correlated with qHBsAg (r=-0.52; p=0.02). pgRNA was also highly negatively correlated with qHBsAg (r=-0.49l; p=0.03) but not HBV DNA (r=0.13; p=0.59). (Figure).

**Conclusion:**

HBV pgRNA is a better predictor of ALT change – a key measure of hepatocyte injury – than HBV DNA in PLWH who are initiated on nucleotide/nucleoside therapy. Quantitative HBsAg is inversely associated with ALT levels, supporting the hypothesis that HBsAg inhibits immune mediated injury to hepatocytes.

**Disclosures:**

**Michael E. Stec, MSc**, Abbott Laboratories: Employee of Abbott|Abbott Laboratories: Stocks/Bonds **mark C. Anderson, PhD**, Abbott Laboratories: Employment|Abbott Laboratories: Stocks/Bonds **Marion G. Peters, MD**, Aligos: Advisor/Consultant|antios: Advisor/Consultant|Excision Biotherapeutics: DSMB **Gavin Cloherty, PhD**, Abbott: Employee|Abbott: Stocks/Bonds|Abbott Labs: Employee.

